# Regulatory Role of Fatty Acid Metabolism-Related Long Noncoding RNA in Prostate Cancer: A Computational Biology Study Analysis

**DOI:** 10.1155/2023/9736073

**Published:** 2023-02-14

**Authors:** Yutao Wang, Hao Su, Yi Lu, Hongjun Li

**Affiliations:** Department of Urology, Chinese Academy of Medical Sciences, Peking Union Medical College, Peking Union Medical College Hospital, Beijing, China

## Abstract

In elderly men, prostate cancer is a leading cause of death. Tumor cells require more energy to progress than normal cells, and this energy is mainly dependent on the large amount of ATP support generated by lipid metabolism. Therefore, in this study, we focused on long noncoding RNAs related to lipid metabolism in prostate cancer to discover the biological mechanisms of lipid metabolism regulation. The TCGA-PRAD cohort was used in this study for computational biology analysis. In lipid metabolism biological pathways, 1959 long noncoding RNAs were identified by Pearson correlation coefficient analysis of protein-coding genes, then univariate regression with *P* values fewer than 0.05. We further identified 784 lncRNAs that were lipid metabolism-related lncRNAs considered to have prognostic value for disease-free survival. Subsequently, we constructed two lncRNA expression patterns of lipid metabolism based on these lncRNAs by nonnegative matrix dimensionality reduction. These two expression patterns showed significant differences in disease-free survival curves for those diagnosed with prostate cancer. We found significant differences in mRNA surveillance pathway and mRNA processing between C1 and C2 groups based on the WGCNA method to explore the biological characteristics of these two expression patterns. Finally, we constructed a disease-free survival (PFS) model based on these lncRNAs. The results identified lncRNAs involved in lipid metabolism and revealed differences in their expression patterns. Additionally, the results offer candidate ideas and approaches concerning the precision treatment of prostate cancer by studying lipid metabolism by candidate long noncoding RNAs.

## 1. Introduction

### 1.1. Relationship between Lipid Metabolism and Prostate Cancer

The American Cancer Society estimates that American men are most likely to suffer from prostate cancer [1] (PCa) in 2022, making up 27% of cancer diagnoses among men. Moreover, the available data indicate that the highest prostate cancer incidence rates are in Europe and the United States, whereas they are lower in Asia. With the development of PSA detection technology, the incidence rate of prostate cancer is declining or stable in nature [2]. In lipid metabolism, lipids are synthesized and degraded inside cells. It has been shown in many studies that lipid metabolism is closely related to the progression of prostate cancer. The management of prostate cancer based on lipid metabolism has become a research hotspot [3, 4]. Abnormal metabolism is a sign of cancer, and tumorigenesis depends on the reprogramming of cell metabolism. Because cancer cells require nutrients from malnourished environments to meet their energy needs and other carcinogenic abilities5, like most other tumors, PCa also undergoes metabolic reprogramming. Prostate cancer cells can increase de novo lipogenesis and fatty acid oxidation by up-regulating androgen receptor (AR)-regulated lipogenic enzymes [5]. Several transcription factors regulate lipogenesis, including sterol regulatory element-binding proteins (SREBPs). AR-mediated interaction between androgens and SREBPs can improve the activation and expression of SREBPs [6]. At the same time, SREBPs can also trigger the AR pathway by activating AR gene expression. It appears that there is a correlation between the two pathways. Moreover, some genetic abnormalities related to PCa also strengthen lipid metabolism. Some studies have shown that the loss or inhibition of p53 activity can reduce androgen receptor-mediated signal transduction in PCa cell lines and inhibit the progression of prostate cancer [7]. As a consequence of the loss of the tumor suppressor gene PTEN, prostate cancer cells undergo metabolic reprogramming, resulting in fatty acid synthase (FAS) overexpression, the production of fatty acids and cholesterol, and the proliferation of prostate cancer cells that are malignant. [8]. Some studies have shown that the overexpression of oncogene MYC can lead to the imbalance of lipid metabolism, induce the expression of fatty acid synthase, and then promote the progression of prostate cancer [9, 10]. Therefore, lipid metabolism plays a vital function in the formation and spread of prostate cancer.

A long noncoding RNA (lncRNA) is a transcript longer than 200 nucleotides with little or no protein-coding potential. There are many processes occurring within cells controlled by lncRNAs, including cell proliferation, metastasis, differentiation, and apoptosis [11]. Localization determines whether lncRNAs are nuclear or cytoplasmic. Nuclear lncRNAs could modulate gene transcription. As a result of chromatin structure regulation, cytoplasmic lncRNAs could regulate mRNA through the interaction between RNAs [12]. SREBPs are crucial in regulating lipid homeostasis by modulating cholesterol and fatty acid metabolism. The SREBP family consists of three subtypes: SREBP1a, SREBP1c, and SREBP2. In many studies, lncRNAs have been shown to contribute to lipid metabolism affecting the biosynthesis of cholesterol and triglycerides, lipid uptake and efflux, cholesterol transport, and other pathways [13]. Some studies have shown that the overexpression of LncARSR, a recently discovered lncRNA, promotes liver cholesterol biosynthesis by increasing the expression of 3-hydroxy-3-methyl-glutaryl coenzyme A reductase (HMGCR), the rate-limiting enzyme for cholesterol synthesis. A possible mechanism by which LncARSR increases HMGCR15 expression is through SREBP-2, the primary transcription factor of HMGCR [14]. Another study identified that a new lncRNA, lncHR1, was negatively related to the expression of SREBP-1c. LncHR1 overexpression inhibits SREBP-1c and fatty acid synthase expression in hepatocytes, inhibiting triglyceride and lipid droplet accumulation caused by oleic acid [15]. In cancer research, nuclear paraspeckle assembly transcript 1 (NEAT1), a nuclear-enriched lncRNA, appears to be overexpressed in various cancerous tissues, and the dysregulation of NEAT1 contributed to cancer progression and metastasis [16]. It has been shown that NEAT1 regulates lipid metabolism via adipose triglyceride lipase (the main enzyme involved in lipolysis), thereby promoting hepatocellular carcinoma [17]. LncRNA MALAT1 (metastasis associated with lung adenocarcinoma transcript 1) has been related to many diseases. It has been found that MALAT1 can induce liver lipid accumulation and insulin resistance via increasing SREBP-1c and target gene expression [18]. Besides regulating key transcription factors for lipid metabolism, lncRNAs can also do so in multiple ways. HULC is a lncRNA that is upregulated in hepatocellular carcinoma. Studies have shown that in hepatoma cells, HULC activates the promoter of Acyl-CoA synthetase long-chain family member1 (ACSL1) by upregulating the transcription factor PPARA, thus regulating lipid metabolism and promoting the proliferation of hepatoma cells [19]. LNMICC (a kind of lncRNA) has been found to reprogramme fatty acid metabolism, recruit nuclear factor NPM1 to FABP5 promoter, and promote cervical cancer lymph node metastasis [20]. Therefore, it appears that lncRNAs regulate lipid metabolism in a major way.

With the development of computational biology, it is necessary to use bioinformatics technology to study the progress of cancer. At present, there are a variety of methods and means for the research of computational biology [21, 22]. Many scholars construct prognostic models of multiple genes for prognostic analysis and screening of prognostic biomarkers. Bioinformatics studies demonstrated that lncRNAs contribute to the progression of many cancers. Cell processes can be influenced by the imbalance of lncRNAs, including proliferation, apoptosis, angiogenesis, and tumor cell metastasis. LncRNAs can also affect the biological progress of cancer through chromatin remodeling and chromatin interaction [23, 24]. Bioinformatics software has been used to investigate prostate cancer through the regulatory networks of lncRNA, miRNA, and mRNA, as well as to examine the genes and pathways involved in PCa pathogenesis [25]. Some studies have established a model that relies on lipid metabolism-related lncRNAs to predict breast cancer patients' survival rates and prognoses, a crucial tool in assessing survival for breast cancer patients [26]. Some studies have developed a contrasting endogenous RNA network (ceRNA) concerning fatty acid metabolism in colorectal cancer (CRC) using bioinformatics methods, identified the lncRNA related to fatty acid metabolism in the ceRNA network pertaining to survival, and constructed a nomogram of the outlook for patients with colorectal cancer [27]. The study was conducted using bioinformatics methods to identify lncRNAs related to lipid metabolism in prostate cancer and constructed a prognostic model of prostate cancer, which is of great significance in guiding prostate cancer management.

## 2. Materials and Method

### 2.1. Data Collection

Transcriptome expression data and clinical information following data for prostate adenocarcinoma in The Cancer Genome Atlas (TCGA) were obtained from the GDC Data Portal (https://portal.gdc.cancer.gov/). TPM data from TCGA-PRAD were used in this analysis. Due to the smaller number of overall survival adverse events samples, we used disease-free survival status as the follow-up endpoint of the investigation. The prostate samples totaled 552 in total, including 500 tumor samples and 52 adjacent carcinoma samples.

### 2.2. Fatty Acid Metabolism Dataset

We downloaded the fatty acid metabolism dataset from the GSEA database (GSEA (https://gsea-msigdb.org)). 42 protein-encoding genes were identified as part of the fatty acid metabolism pathway. The long noncoding RNAs related to the fatty acid metabolism pathway were determined by a Pearson coefficient of more than 0.4.

### 2.3. Univariate Cox Regression

Cox models can be used to examine the effect of several elements on survival time when survival outcome and survival time are the indicators of dependency simultaneously. Data with censored survival times can also be examined without needing the estimation of survival distributions. As a result of these excellent properties, long noncoding RNA survival assessment was conducted using multivariate COX regression.

### 2.4. Fatty Acid Metabolism-Related lncRNAs Expression Patterns

We performed nonnegative matrix dimensionality reduction (NMF) on the relevant human coding genes for the long noncoding RNAs identified previously. Before NMF analysis, we preprocessed the data. First, candidate genes with an absolute median (MAD) of less than 0.5 were excluded. Genetic association of all candidates relating to overall survival was then assessed using Cox regression. The “survival” package was applied for analysis. Finally, the absolute median was more significant than 0.5; genes of 0.05 were used for nonnegative matrix factorization. This was performed through the “NMF” [5] R package.

### 2.5. WGCNA Analysis

An analysis of coexpression was conducted using WGCNA to develop a model related to C1 and C2 models. In general, gene sets with the same expression pattern tend to have similar expression profiles, and these functionally equivalent genes constitute intricately linked coexpression networks. Therefore, we determined the coexpression network between the expression patterns of long noncoding RNAs related to different fatty acid metabolism using coexpression identification on TCGA-PRAD. We first performed a sample cluster analysis on the TCGA-PRAD cohort. The weighted coexpression network was created with the R language WGCNA package [28]. To calculate the soft threshold for the upcoming network construction, the most appropriate weighted parameters of the adjacent functions were calculated using pickSoftThreshold. Based on the hierarchical clustering of the dissimilarity measure of the topological overlap matrix, we constructed the weighted adjacency matrix as well as the associated gene modules (TOM) (1-TOM) [29]. Finally, the Pearson correlation coefficient was calculated between different coexpression modules and cluster categories.

### 2.6. Functional Enrichment Analysis

To explain biological differences between different fatty acid metabolism-related long noncoding RNA expression patterns, we performed functional analysis on protein-coding genes in the coexpression module. Functional enrichment analysis, including gene ontology (GO) analysis and KEGG pathway enrichment of the coexpression genes, was carried out with the “clusterProfiler” [30] and “pathview” packages [31].

### 2.7. Immune-Inflammatory Response

Multiple gene sets able to represent the immune response were tested for their role in the immune microenvironment of prostate cancer involving long noncoding RNA (lncRNA). These gene sets included significant histocompatibility complex class II (MHC-II), lymphocyte-specific kinase (LCK), hematopoietic cell kinase (HCK), immunoglobulin G (IgG), signal transducer and activator of transcription 1 (STAT1), costimulatory molecule (B7-CD28), interferon, and TNF gene sets [32]. An algorithmic tool called ESTIMATE (using expression data to estimate stromal and immune cells in malignant tumor tissue) was used to analyze tumor purity [33].

## 3. Results

### 3.1. Research Technical Route

**Figure 1:**
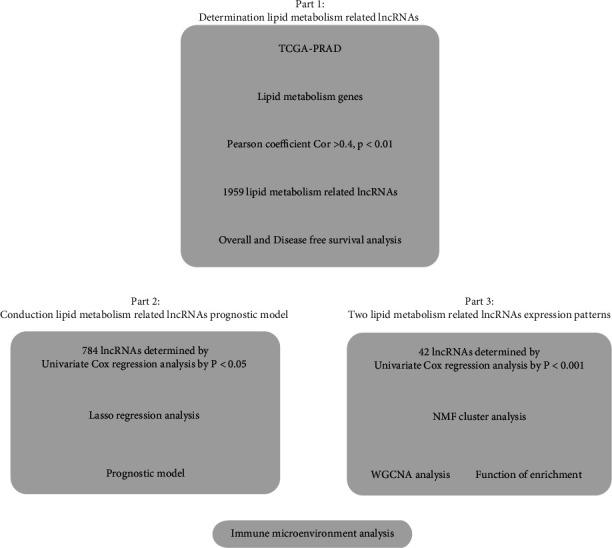
Flowchart.

The research route of this paper is uploaded in Figure 1. There are primarily three aspects to the research. In the first aspect, noncoding RNAs associated with fatty acid metabolism are identified in prostate cancer. The second part constructs the prognosis model of long noncoding RNA related to fatty acid metabolism in prostate cancer. The third aspect looks at different expression patterns of two long noncoding RNAs related to fatty acid metabolism, and the biological differences between them were explored.

### 3.2. Identification of Fatty Acid Metabolism-Related Long Noncoding RNAs

**Figure 2:**
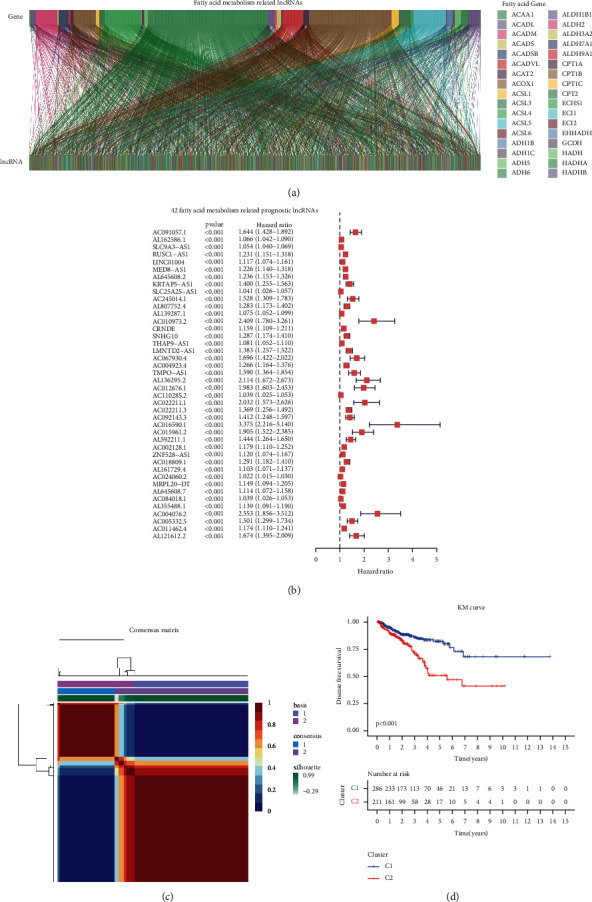
Determination of two fatty acid metabolism lncRNAs expression patterns. (a) Fatty acid metabolism-related lncRNAs. (b) 42 fatty acid metabolisms related lncRNAs with *P* < 0.001. (c) Two fatty acid metabolism lncRNAs expression patterns. (d) Disease-free survival analysis between deference lncRNAs expression patterns.

According to the *P* < 0.01, Cor <0.4, we identified 1,959 long noncoding RNAs **(**Figure 2(a)**)**. We then evaluated the prognostic value of the above 1,959 lncRNAs independently, with disease-free survival status as the end point of follow-up. In univariate Cox regression, we screened 784 lncRNAs associated with fatty acid metabolism. A univariate Cox regression with a P 0.05 was used as the screening criterion. The hazard ratios and confidence intervals for the most significant 42 lncRNAs are shown in Figure 2(b). Subsequently, we performed a cluster analysis based on the above 42 long noncoding RNAs related to fatty acid metabolism. Our clustering results revealed two types of expression patterns for long noncoding RNA (Figure 2(c)). In the survival follow-up curve of the disease-free survival state, patients with two different lncRNA expression patterns displayed significant prognostic disparities, *P* < 0.001**(**Figure 2(d)**).**

### 3.3. Identification of Biological Differences between Different Fatty Acid Metabolisms Based on Long Noncoding RNA Expression Patterns

**Figure 3:**
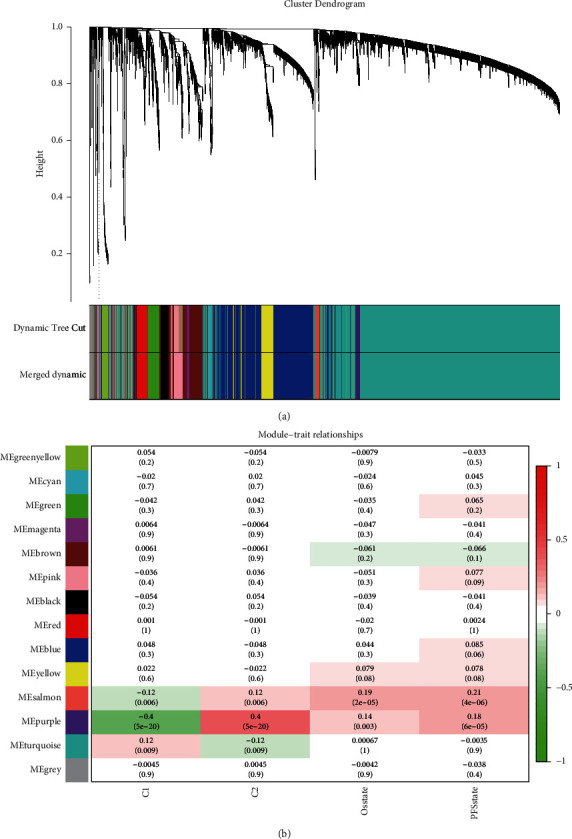
WGCNA analysis. (a) Cluster dendrogram. (b) WGCNA coexpression modules related to the C2 cluster, and the correlation coefficient between the purple module and the C2 cluster is 0.40.

**Figure 4:**
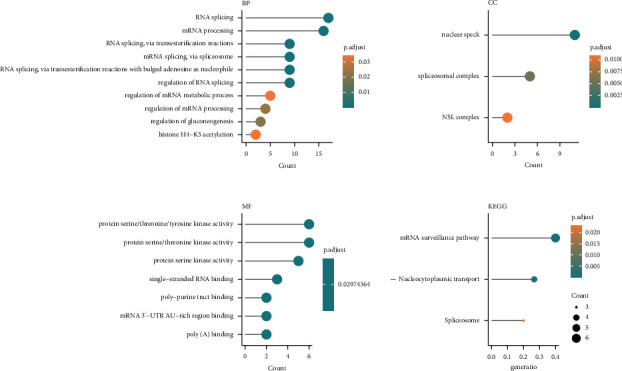
The GO and KEGG analysis of the purple coexpression protein-coding genes.

Using lncRNAs involved in fatty acid metabolism, we identified two different expression patterns. To determine the biological differences between the two expression patterns, we constructed coding gene coexpression modules of the two expression patterns using the WGCNA method. Protein-coding genes and clinical samples of prostate cancer were included in the WGCNA entry file, and samples with similar expression patterns were included in the subsequent analysis based on omics cluster analysis. According to the cut-off value of 80000, 492 prostate cancer samples with similar expression patterns were obtained. We set the *β* value to 5, the gene in the minimum module to 30, and finally obtained 14 coexpression modules **(**Figures 3(a) and 3(b)**)**. We found that the long-chain noncoding trait group of C2 fatty acid metabolism was most strongly associated with the purple module **(**Figure 3(b); Cor = 0.40). The purple module contained 49 genes. The genes with a correlation greater than 0.4 with the C2 group in the purple module were functionally enriched, and the findings indicated their involvement in RNA splicing, mRNA processing, regulation of RNA splicing, and mRNA surveillance pathway and other biological processes **(**Figure 4**)**.

### 3.4. To Construct a Prognostic Model of Prostate Cancer Disease-Free Survival Based on Long Noncoding RNA Related to Fatty Acid Metabolism

**Figure 5:**
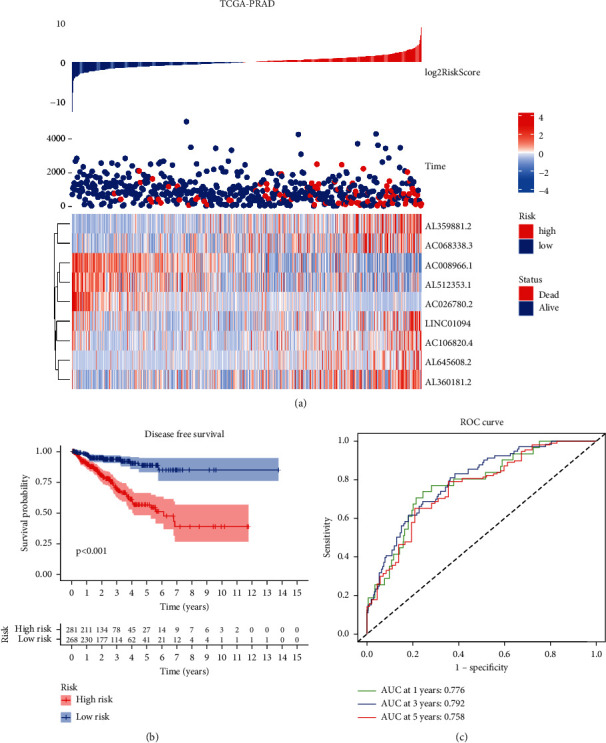
The clinical presentation of the prognostic risk signature in the TCGA-PRAD set. (a) The distribution of the risk score and disease-free survival (DFS) status in high- and low-risk groups. (b) Results of the Kaplan–Meier analysis showed that the high-risk group had worse DFS rates in the TCGA-PRAD. (c) ROC diagnostic test.

A training set was randomly selected from the TCGA-PRAD cohort, followed by a validation set. The samples were sorted according to their IDs, and random numbers were assigned using SPSS to each to enable categorization. Using Lasso regression, we constructed a prognostic model for long noncoding RNA related to fatty acid metabolism. We took the long noncoding RNA related to fatty acid metabolism as the entry data. Based on the disease-free survival rate as clinical follow-up data, regression analysis was performed. With so many genes, it is difficult to conduct clinical identification. The R package glmnet was used to perform Lasso regression evaluation to limit the scope of long noncoding RNA and maintain high accuracy. Finally, we constructed a 9-gene prognostic risk model. RiskScore = 0.71^*∗*^ expAC106820 + 0.20^*∗*^ expAL359881 + 0.12^*∗*^ expAL645608 − 1.55^*∗*^ expAC026780 + 0.79^*∗*^expLINC01094 + 0.20^*∗*^ expAC068338 − 0.62^*∗*^ expAC008966 − 0.62^*∗*^ expAL512353 + 0.16^*∗*^ expAL360181. We evaluated the RiskScore according to the TPM value of long noncoding RNA and the corresponding risk coefficient. We obtained the sample distribution by RiskScore, as shown in Figure 5(a). There is a substantial difference between adverse event samples with low and high RiskScores, indicating that adverse event samples with high RiskScores may be at increased risk. The KM curve is shown in Figure 5(b) after we separated RiskScores into low- and high-risk groups. There was a significant survival difference between high- and low-risk scores (*P* < 0.001). Immediately after, we used ROC to evaluate RiskScores. We analyzed the 1.3- and 5-year predictive classification efficiency, and the model is shown in Figure 5(c), with an AUC area of 0.758 of 5 years, 0.792 of 3 years, and 0.776 of 1 year **(**Figure 5(c)**)**.

### 3.5. Evaluation of Prognostic Models for Long Noncoding RNAs

**Figure 6:**
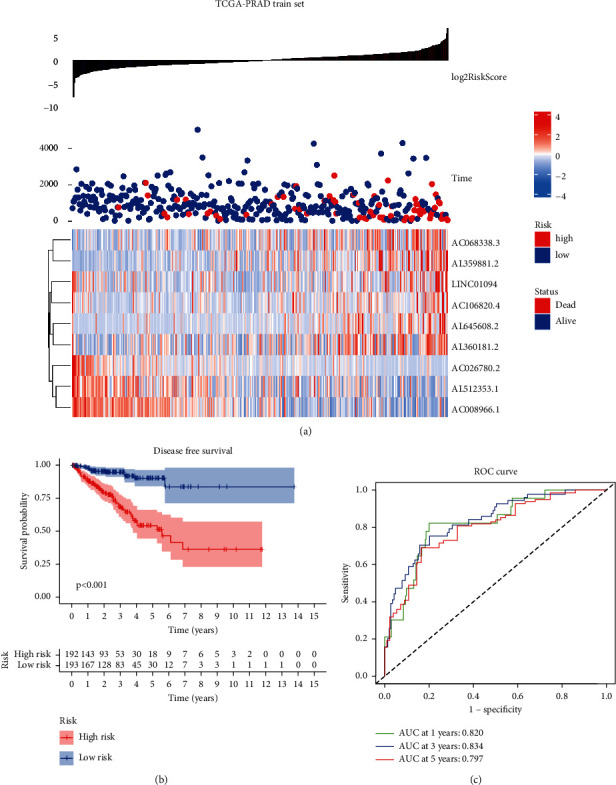
The clinical presentation of the prognostic risk signature in the train set. (a) The distribution of the risk score and disease-free survival (DFS) status in high- and low-risk groups. (b) Results of the Kaplan–Meier analysis showed that the high-risk group had worse DFS rates in the train set. (c) ROC diagnostic test.

**Figure 7:**
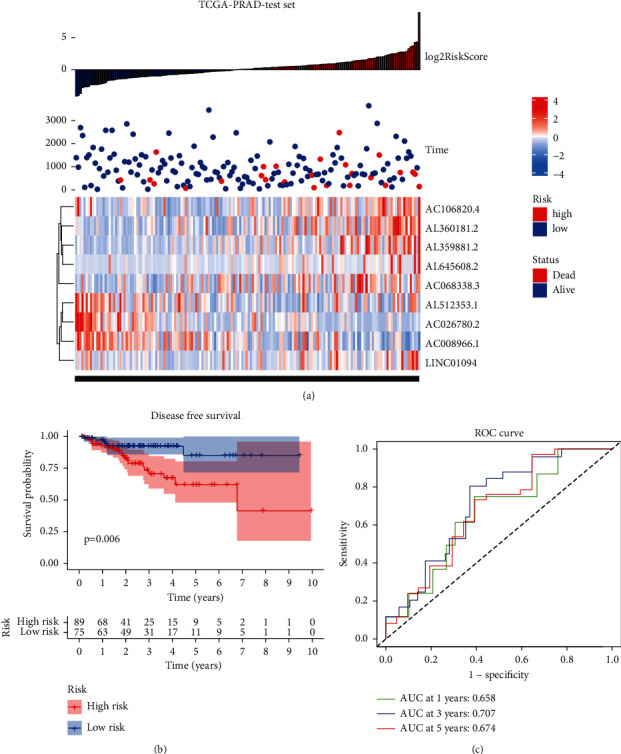
The clinical presentation of the prognostic risk signature in the test set. (a) The distribution of the risk score and disease-free survival (DFS) status in high- and low-risk groups. (b) Results of the Kaplan–Meier analysis showed that the high-risk group had worse DFS rates in the test set. (c) ROC diagnostic test.

In addition, we analyzed the training and test datasets for the association between risk scores and long noncoding RNAs. According to the results of the TCGA-PRAD training set, patients with high-risk scores had a less favorable outcome than those with low-risk scores, *P* < 0.001. The ROC experimental diagnostic efficiency of the risk score model in the training set for disease-free recurrence was 0.797 in the 5-year AUC area. The 3-year AUC area was 0.834, and the 1-year AUC area was 0.820 **(**Figure 6**)**. At the same time, the prognosis of patients in the high-risk score group was worse than that in the low-risk score group in the validation set, *P* < 0.001. The ROC experimental diagnostic performance of the risk scoring model for disease-free recurrence in the training set was 0.674 in 5 years, 0.707 in 3 years, and 0.658 in 1 year **(**Figure 7**)**.

### 3.6. Relationship between LINC01094 and Immune Microenvironment

**Figure 8:**
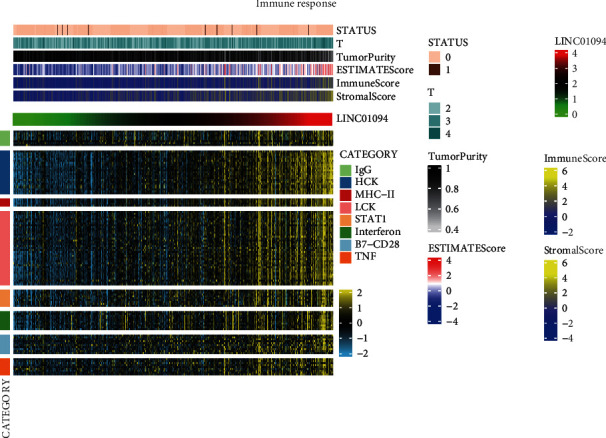
The correlation between immune-inflammation response and LINC01094.

In our study, we found that LINC01094 was an independent prognostic in prostate cancer disease-free survival. LINC01094 is in conjunction with a poor prognosis, in various cancer types, but its effect on the prostate cancer microenvironment is still unclear. As a result, this analysis examines the significance of LINC01094 in prostate cancer's tumor microenvironment through various immune validation response gene sets. An increase in inflammation and immune responses was positive in association with LINC01094. These reactions were triggered by hematopoietic cell kinases, immunoglobulin *G*, interferon, lymphocyte-specific kinase, primary histocompatibility complex class I, major histocompatibility complex class II, and activator of transcription 1. This evidence indicated that patients with elevated LINC01094 also displayed more clusters of immune inflammation **(**Figure 8**)**.

## 4. Discussion

Among men, prostate cancer is the second most common cancer-related death and the predominant malignancy of the urinary system. Moreover, prostate cancer has evident heterogeneity, and its incidence rate and mortality vary significantly in different regions and ethnic groups, which also brings considerable challenges to treating prostate cancer [34]. For individualized treatment, it is vital to discover prognostic biomarkers and understand the molecular mechanism of prostate cancer. Studies have discovered a strong connection between lipid metabolism disorders and prostate cancer. The change in fatty acid synthesis is a distinct feature of prostate cancer and a treatment target [35]. However, there is still a minimal amount of data on the connection between lipid metabolism and lncRNA in prostate cancer.

Based on fatty acid metabolism pathway genes, we identified long-chain noncoding RNA genes affecting fatty acid metabolism pathway genes. Patients with prostate cancer are further clustered using RNA. In patients with different expression patterns of long noncoding RNAs related to fatty acid metabolism pathway genes, survival and disease-free survival showed significant differences. Then, the fatty acid metabolism pathway genes related to long-chain noncoding RNA are used to build the disease-free survival of the prostate cancer prognosis model. The model includes multiple variables, such as AC106820 AL645608, AC026780, LINC01094, AC068338, AC008966, AL51235, and AL360181.

LINC01094 is a novel lncRNA. According to studies, LINC01094 has an essential function in the advancement and invasion of several cancer types. There is a high expression of LINC01094 in clear cell renal cell carcinoma (ccRCC). LINC01094 acts as a competitive endogenous RNA in ccRCC and plays a tumor-promoting contribution to the progress of ccRCC through the microRNA 224-5p/chondroitin synthase 1 (CHSY1) regulatory axis [36]. Other studies have found that LINC01094 is significantly upregulated in ovarian cancer (OC) tissues and cells. LINC01094 can promote the proliferation, migration, and invasion of OC cells by downregulating miR-532-3p [37]. In addition, further studies have found that LINC01094 is overexpressed in pancreatic cancer. LINC01094 regulates lin-28 homolog B (LIN28B) expression and PI3K/AKT pathway serving as a ceRNA of miR-577, which promotes the proliferation and metastasis of pancreatic cancer [38]. The expression of LINC01094 in glioma tissues and cell lines is highly correlated with high-grade gliomas, according to some studies. A miR-330-3p/MSI1 axis is regulated by LINC01094 to promote glioma cell proliferation, migration, and invasion [39]. Other studies have found that LINC01094 is more abundant in breast cancer tissues than in normal tissues. There is a correlation between high LINC01094 expression and shorter overall survival for breast cancer patients. LINC01094 induces cell cycle progression by regulating the microRNA-340-5p (miR-340-5p)/E2F transcription factor 3 (E2F3) molecular axis, as a result, facilitating the spread and progression of breast cancer cells [40]. Some studies have found that LINC01094 is highly expressed in gastric cancer tissues and is an independent index to estimate adverse survival rate. LINC01094 could participate in epithelial-mesenchymal transition (EMT) and tumor-associated macrophages (TAMs) infiltration of gastric cancer to promote the progression and metastasis of gastric cancer [41]. In addition, studies have found that LINC01094 is highly expressed in colorectal cancer cells. LINC01094 can promote the progression of colorectal cancer by regulating the miR-1266-5p/secretory leukocyte protease inhibitor (SLPI) axis [41]. Therefore, LINC01094 can function as an oncogenic factor that may accelerate cancer progression across numerous cancer types.

Our research is of great significance. It reveals the regulatory role of lncRNAs in the lipid metabolism of prostate cancer and introduces a novel direction for the prognosis and treatment of prostate cancer. But there are still many limitations to this study. First, all prostate cancer information comes from the TCGA database, in which the patients are primarily American. There are no prostate cancer patients from other regions and countries. Second, the lncRNAs-related mechanism of lipid metabolism in prostate cancer needs further in vivo and in vitro experiments to clarify as well as further experimental research.

## Data Availability

The original data of the samples in this study can be obtained from the open-source database TCGA.

## Abbreviations

NMF: Nonnegative matrix factorization
WGCNA: Weighted correlation network analysis
Lasso: Least absolute shrinkage and selection operator
GSEA: Gene set enrichment analysis
ESTIMATE: Estimation of stromal and immune cells in malignant tumor tissues using expression data.
